# The Role of Cardiac T-Cadherin in the Indicating Heart Failure Severity of Patients with Non-Ischemic Dilated Cardiomyopathy

**DOI:** 10.3390/medicina56010027

**Published:** 2020-01-09

**Authors:** Vaida Baltrūnienė, Ieva Rinkūnaitė, Julius Bogomolovas, Daiva Bironaitė, Ieva Kažukauskienė, Egidijus Šimoliūnas, Kęstutis Ručinskas, Roma Puronaitė, Virginija Bukelskienė, Virginija Grabauskienė

**Affiliations:** 1 Department of Pathology, Forensic Medicine and Pharmacology, Faculty of Medicine, Vilnius University, 01513 Vilnius, Lithuania; ieva.zasytyte@gmail.com (I.K.); virginija.grabauskiene@santa.lt (V.G.); 2 Institute of Biochemistry, Life Sciences Center, Vilnius University, 01513 Vilnius, Lithuania; ieva.rinkunaite@gmail.com (I.R.); Egidijus.simoliunas@gmc.vu (E.Š.); virginija.bukelskiene@bchi.vu.lt (V.B.); 3 Department of Medicine, University of California, San Diego, CA 92093, USA; jbogomolovas@ucsd.edu; 4 Department of Regenerative Medicine, State Research Institute, Center for Innovative Medicine, 08410 Vilnius, Lithuania; daibironai@gmail.com; 5 Center of Cardiac Surgery, Clinic of Cardiac and Vascular Diseases, Faculty of Medicine, Vilnius University, 01513 Vilnius, Lithuania; kestutis.rucinskas@santa.lt; 6 Clinic of Cardiac and Vascular Diseases, Institute of Clinical Medicine, Faculty of Medicine, Vilnius University, 01513 Vilnius, Lithuania; roma.puronaite@gmail.com; 7 Institute of Data Science and Digital Technologies, Faculty of Mathematics and Informatics, Vilnius University, 01513 Vilnius, Lithuania

**Keywords:** adiponectin, advanced heart failure, non-ischemic dilated cardiomyopathy, T-cadherin

## Abstract

*Background and objectives*: T-cadherin (T-cad) is one of the adiponectin receptors abundantly expressed in the heart and blood vessels. Experimental studies show that T-cad sequesters adiponectin in cardiovascular tissues and is critical for adiponectin-mediated cardio-protection. However, there are no data connecting cardiac T-cad levels with human chronic heart failure (HF). The aim of this study was to assess whether myocardial T-cad concentration is associated with chronic HF severity and whether the T-cad levels in human heart tissue might predict outcomes in patients with non-ischemic dilated cardiomyopathy (NI-DCM). *Materials and Methods:* 29 patients with chronic NI-DCM and advanced HF were enrolled. Patients underwent regular laboratory investigations, echocardiography, coronary angiography, and right heart catheterization. TNF-α and IL6 in serum were detected by enzyme-linked immunosorbent assay (ELISA). Additionally, endomyocardial biopsies were obtained, and the levels of T-cad were assessed by ELISA and CD3, CD45Ro, CD68, and CD4- immunohistochemically. Mean pulmonary capillary wedge pressure (PCWP) was used as a marker of HF severity, subdividing patients into two groups: mean PCWP > 19 mmHg vs. mean PCWP < 19 mmHg. Patients were followed-up for 5 years. The study outcome was composite: left ventricular assist device implantation, heart transplantation, or death from cardiovascular causes. *Results:* T-cad shows an inverse correlation with the mean PCWP (rho = −0.397, *p* = 0.037). There is a tendency towards a lower T-cad concentration in patients with more severe HF, as indicated by the mean PCWP > 19 mmHg compared to those with mean PCWP ≤ 19 mmHg (*p* = 0.058). Cardiac T-cad levels correlate negatively with myocardial CD3 cell count (rho = −0.423, *p* = 0.028). *Conclusions:* Univariate Cox regression analysis did not prove T-cad to be an outcome predictor (HR = 1, *p* = 0.349). However, decreased T-cad levels in human myocardium can be an additional indicator of HF severity. T-cad in human myocardium has an anti-inflammatory role. More studies are needed to extend the role of T-cad in the outcome prediction of patients with NI-DCM.

## 1. Introduction

The current concept of heart failure (HF) pathogenesis includes a broad spectrum of structural, functional, electrophysiological, cellular, and molecular abnormalities [1]. Pathological molecular mechanisms, including myocyte loss, cardiac hypertrophy, the alteration of extracellular matrix homeostasis, fibrosis, defective autophagy, metabolic abnormalities, and mitochondrial dysfunction eventually lead to left ventricular remodeling and cardiac dysfunction [2,3]. Patients with advanced HF develop various additional derangements, including changes in adipokine levels, long-term systemic inflammation, and progressive catabolism [4,5].

One such adipokine, which has a critical signaling function in the heart, is adiponectin (APN). At the cellular and molecular levels, APN exerts anti-inflammatory, antioxidant, and antiapoptotic activity [6,7,8,9]. Its anti-inflammatory [10], hypertrophy [11] and fibrosis [12] reducing activity is considered to be important in cardio-protection. Unexpectedly elevated circulating levels of this adipokine have been documented in chronic HF patients [13,14], correlating with increased cardiovascular and all-cause mortality [15,16]. A possible explanation for this paradox might be the diminished activity of the APN/APN receptor system due to changed levels of receptors, reduced receptor sensitivity, or dysfunctional post-receptor signaling [17].

Inflammation is a hallmark of chronic HF [18]. Data from previous studies indicate that low-grade inflammation suppresses the production of APN in adipocytes and might lead to hypoadiponectinemia in metabolic diseases [6,19,20]. Decreased APN levels have been connected to a higher risk of acute MI myocardial infarction in men without cardiovascular disease [21], increased myocardial injury [10], and a higher risk of coronary artery restenosis [22]. However, after the development of HF, serum APN levels seem to be no longer negatively controlled by low-grade inflammation, as reviewed by Van Linthout et al. [18]. Even more, a positive association between inflammation and APN is usually reported in chronic HF [23]. Higher levels of circulating APN are also linked to the poor outcome of patients with chronic HF [24,25,26,27]. It is still questionable if APN loses its anti-inflammatory and cardioprotective activity in chronic HF, or the compensatory increase of APN in serum is unable to counteract the increasing pro-inflammatory milieu [5].

T-cad is a binding molecule for hexameric and high molecular weight APN [28]. Through the association with T-cad, APN seems to diminish pathological cardiac remodeling, promote revascularization, and exert vasculoprotective actions [29,30,31,32,33]. T-cad deficiency mimics APN deficiency in chronic and acute mouse models of cardiac injury [32]. Accumulating data suggest that T-cad might be a critical modulator of APN levels in the blood and tissues: APN levels in T-cad knockout mice plasma were significantly elevated [32,34] and single-nucleotide polymorphisms near the T-cad gene strongly correlated with circulating APN levels in humans [35,36]. Epigenetic changes have also been implicated in the pathogenesis of chronic HF [37,38] as well as in the regulation of myocardial APN receptor expression [39]. S. Kreth and colleagues have demonstrated that microRNA-150 is increased in the myocardium of patients with end-stage chronic HF and is inversely correlated with adiponectin receptor 2 transcript levels.

Several clinical studies have investigated the expression of APN receptors in the myocardium of patients with advanced HF [39,40,41], but to our knowledge, none of them investigated the levels and impact of cardiac T-cad in it. Since the binding of APN with T-cad in the myocardium is a prerequisite in animal models [32], the decrease in myocardial T-cad levels might be related to an increased concentration of circulating APN, and the diminished anti-inflammatory activity of this adipokine.

Despite the improvement in the yearly rate of major adverse cardiovascular events, NI-DCM remains one of the major causes of chronic HF, eventually leading to heart transplantation [42]. The course of the disease is not easy to predict: some patients remain stable, while others deteriorate quickly [43]. Therefore, the search for novel prognostic biomarkers or a combination of markers is essential for improving clinical decision making and transplantation list prioritization.

The aim of this pilot study was to assess whether myocardial T-cad concentration is associated with chronic HF severity and whether the T-cad levels in human heart tissue might predict outcomes in patients with chronic non-ischemic dilated cardiomyopathy (NI-DCM).

## 2. Materials and Methods

### 2.1. Patients

The present study is a part of a previous study run in 2010–2013 in tertiary care, “Vilnius University Hospital Santaros klinikos”, which enrolled patients admitted to a tertiary care cardiology center with a suspected diagnosis of NI-DCM.

The inclusion criteria were as follows: the exacerbation of HF symptoms, accompanied by echocardiographic evidence of dilated cardiomyopathy: (1) left ventricular end-diastolic diameter (LVEDD) > 117% (>2 SD of the predicted value of 112% corrected for age and body surface area) (2) left ventricular systolic dysfunction, defined by left ventricular ejection fraction (LVEF) < 45% (>2 SD), and/or (3) fractional shortening < 25% (>2 SD). The patients were excluded in case of significant coronary artery disease (>50% stenosis on coronary angiography) or myocardial infarction in the history and the presence of acute myocarditis or other heart diseases, including primary valvular heart disease, toxic DCM, arterial hypertension, as well as diabetes, renal disease, and the abuse of alcohol or illicit drugs.

NI-DCM patients: 57 subsequent patients were enrolled. All of them underwent a careful history and physical examination, routine laboratory studies, echocardiography for an LV function evaluation, coronary angiography for the exclusion of coronary artery disease, and a right heart catheterization for hemodynamic evaluation. An endomyocardial biopsy (EMB) was performed for the assessment of inflammation as the primary cause of otherwise unexplained dilated cardiomyopathy as well as virus genome evaluation with polymerase chain reaction. CD3, CD45Ro, CD68, CD4, CD54, and HLA-DR count in EMB samples was evaluated by immunohistochemistry. All patients were treated according to the guidelines for the Diagnosis and Treatment of Acute and Chronic Heart Failure of the European Society of Cardiology [44].

Patients were followed-up for at least 5 years after inclusion in the study. A median of follow-up was 59 months (min. 14 days, max. 94 months). The first date of the follow-up was the date of the EMB biopsy. The study outcome was a composite consisting of three possible endpoints: left ventricular assist device (LVAD) implantation, heart transplantation (HT), or death from cardiovascular causes. Those states meant either death or a very severe cardiac state for the patient with exhausted therapeutic measures. The time of the first event was the time of the endpoint. Follow-up data were updated once a year (the exact date of the event was included in statistical analysis). Most data concerning endpoint occurrence were retrieved from clinical records and from the national death registry. Only several patients, who did not attend our outpatient clinic regularly, were interviewed by phone calls. Two patients were lost to follow-up after having participated in the study for three years. The clinical characteristics of these patients are presented in our previous paper [45].

Sample size calculation of the present study. Because of the lack of relevant data concerning the concentration of T-cad in the myocardium of patients with chronic NI-DCM, it was difficult to choose the exact standardized effect size. The preliminary sample size was determined following the rule of thumb by Kieser and Wassmer. This rule suggests that a pilot trial sample between 20 and 40 minimizes the overall sample size for a main study sample size of 80–250, corresponding to standardized effect sizes of 0.4 and 0.7 (for 90% power based on standard sample size calculation). From the cohort of 57 patients, suitable previously collected EMB samples of 29 patients were used for the subsequent investigation of cardiac T-cad. Those 29 patients comprise the cohort of the present study.

**Patient groups**: In order to compare cardiac T-cad expression levels in patients with different HF severity, patients were divided into two groups based on their mean pulmonary capillary wedge pressure (PCWP) value. Mean PCWP, a surrogate marker of LV end-diastolic pressure, was chosen as a biomarker of HF severity for patients with chronic NI-DCM and advanced HF. The most appropriate mean PCWP cut-off point was found building a recursive partitioning survival tree using party package in R: patients with a mean PCWP > 19 mmHg had a significantly worse composite outcome compared to those with a mean PCWP < 19 mmHg (*p* = 0.003). This cut-off value was used for the subsequent analysis.

### 2.2. Biochemical Assays of APN and Other Serological Markers

Blood samples data were obtained on the same day as the cardiac catheterization and EMB data. The pro-inflammatory serum cytokines, TNF-α and IL-6, were measured by solid-phase, chemiluminescent immunometric assays using IMMULITE/Immulite 1000 systems (Immulite, Siemens, Munich, Germany) according to the manufacturer’s instructions: TNF-α (Catalog No: LKNFZ (50 tests)), LKNF1 (100 tests); IL-6 (Catalog No: LK6PZ (50 tests)), LK6P1 (100 tests). APN was measured by a Millipore Adiponectin assay according to the manufacturer’s recommendations (Millipore, Burlington, MA, USA).

Brain natriuretic peptide (BNP) in plasma was measured with a two-step immunoassay using chemiluminescent microparticle immunoassay (CMIA) technology and protocols referred to as Chemiflex.

### 2.3. Echocardiography

Echocardiography was performed after admission by an investigator blinded for the study objectives. The standard views were acquired between 70 and 90 frames/s. Conventional echocardiographic parameters such as LVEF, LV end-diastolic diameter (LVEDD), and global strain were evaluated. The methodology for the quantification of echocardiographic parameters is presented in detail in our previous paper [45].

### 2.4. Cardiac Catheterization and EMB

Written, informed consent for cardiac catheterization, including EMB and coronary angiography, was signed by all patients enrolled in the study. Coronary angiography was done for all participants to exclude significant coronary artery disease (stenosis > 50%). Each patient underwent right heart catheterization in order to evaluate hemodynamic impairment and to confirm pulmonary hypertension (PH) diagnosis. Intracardiac hemodynamic parameters, including the mean pulmonary artery pressure (PAP), right atrial pressure (RAP), PCWP, pulmonary vascular resistance (PVR), and cardiac output (CO), were assessed.

EMBs were collected using a flexible bioptome (Westmed) via the right femoral vein. Biopsies were drawn from the right inter-ventricular septum. At least 3 EMBs were subjected to conventional histologic and immunohistochemical evaluation, and 2 EMBs were stored as retained bio-samples. They were promptly placed in −70 C and were investigated within 24 h.

### 2.5. Histological and Immunohistochemical Assessment of EMBs

EMB samples for histological analysis were fixed in 10% buffered formalin and subsequently paraffin-embedded in a tissue processor. Dallas criteria were used for histological diagnosis. Autoantibodies (Santa Cruz Biotechnology, Inc., Dallas, TX, USA) against CD3 (DAKO A0452 Rabbit 1, Hamburg, Germany), CD45Ro (DAKO Hamburg), CD68 (DAKO M0876 Mouse 1, Hamburg, Germany), CD4 (DAKO Hamburg, Germany), CD54 (NovocastraTM Lyophilized Mouse Monoclonal Antibody CD54 Clone 23G12), and HLA-DR (DAKO Hamburg, Germany) were used for immunohistochemical staining. The positively stained cells in the EMB sample were scored by an experienced pathologist and expressed as a number of positive cells/mm^2^. An EMB sample was considered to be inflamed if immunohistochemical staining revealed significant inflammatory cellular infiltrates (≥14 leucocytes/mm^2^, including up to 4 monocytes/mm^2^ with the presence of CD 3 positive T-lymphocytes ≥ 7 cells/mm^2^) [46].

### 2.6. Estimation of T-Cad in Myocardial Biopsies

Stored EMBs were kept at −80 °C. Before the assay, tissue samples were weighed and finely minced 3 × 10 min on ice in appropriate proportions of cold phosphate buffered saline (according to the ELISA kits manufacturer’s recommendations) using a FB15061 (Fisherbrand) sonicator. The samples were then centrifuged at 12,000× *g* for 15 min. The supernatant was used for the assay. The amount of total protein in biopsy samples was estimated by the modified Lowry Protein Assay kit (Thermo Scientific Inc., Waltham, MA, USA). Human T-cad (Cat. No.: abx051689) concentration in EMB was estimated by using commercially available ELISA assay kits (Abbexa, Cambridge, UK). Absorbance was measured at 450 nm with a spectrophotometer (Spectramax^®^i3, Molecular Devises, San Jose, CA, USA). Total protein concentration was expressed as μg/mL. The final concentration of T-cad was expressed as ng/mg of protein. All procedures were performed according to the provided manufacturer’s manuals.

### 2.7. Statistical Analysis

Statistical analysis was performed by the Statistical Package for the Social Sciences (version 23.0 for Windows; IBM.SPSS statistics) and R studio packages (version 1.1.463; 2018 R Studio, Inc., Boston, MA, USA) at a significance level no higher than 5%. In the case of the normal distribution, continuous variables were expressed as means ± standard deviations; otherwise, as medians ± interquartile ranges. Differences in the subgroups of patients were tested with the non-parametric Wilcoxon rank-sum test. For differences between categorical variables, a Chi-squared test or Fisher exact test were used. The association between continuous variables was estimated by the Spearman correlation. One patient with a T-cad value of 161.54 ng/mg was excluded from further analysis as an absolute outlier (z-score 3.45).

Survival tree analysis was performed using the recursive partitioning method for building the decision tree. The root node of the survival tree included 28 patients. The log-rank test was used for node-splitting. The recursive procedure produces offspring nodes until no further statistically significant split is obtained. The decision tree received was binary, and each terminal node represented a group of patients with different survival outcomes depending on the mean PCWP value.

A Kaplan–Meier analysis and log-rank test were used for a comparison of the cumulative survival rates between the subgroups of patients stratified by the median T-cad level. Univariate Cox regression analysis was used to assess the impact of continuous and binary T-cad levels on patient outcomes.

### 2.8. Ethical Approval

The study was approved by the local Lithuanian Bioethics Committee (license No. 158200-09-382-l03; No. 158200-382-PP1-23, No. 158200-17-891-413). Informed consent approved by the Lithuanian Bioethics Committee was signed by all study patients. The study was conducted in accordance with the Declaration of Helsinki.

## 3. Results

### 3.1. Baseline Patient Characteristics

Out of 29 patients with NI-DCM included in the study, 25 (86%) were ranked as NYHA III and IV classes, and all had increased B-type natriuretic protein (BNP) values (Table 1). All patients had long-lasting HF symptoms at the beginning of the study, consistent with chronic and advanced HF.

### 3.2. Relationship Between T-Cad Expression and Other Biomarkers, and the Parameters of Chronic HF

In order to investigate the relevance of cardiac T-cad as a biomarker in patients with NI-DCM and advanced HF, the association with other biomarkers and the parameters of chronic HF was assessed (Table 2). A negative correlation between cardiac T-cad and the mean PCWP is shown (rho = −0.397, *p* = 0.037) in Table 2.

No correlation between the serum APN and the cardiac T-cad expression (rho = −0.027, *p* = 0.892) was found in this study (Table 2), even though it is well described in animal models [31,34]. Correlation analysis demonstrated that a higher T-cad expression is associated with a lower myocardial infiltration of T cells (CD3+) (Table 2).

### 3.3. T-Cad Levels Are Reduced in Patients with More Severe Heart Failure

**Figure 1:**
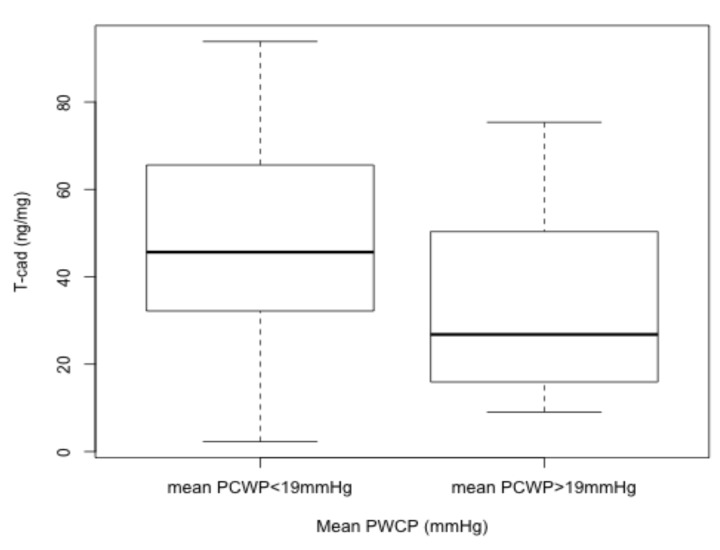
Cardiac T-cad expression based on the mean PCWP.

There is a tendency towards a lower T-cad level in patients with a mean PCWP > 19 mmHg group (*p* = 0.058), as shown in Figure 1. T-cad concentration is diminished in more severe cases of HF concomitantly with decreased LVEF, LV average global strain, and higher BNP values that indicate disease severity (Table 3). A strong association between the mean PCWP and other, already established, biomarkers of HF (LVEF, BNP and LV average global strain) is demonstrated in Table 3.

Patients with a mean PCWP > 19 mmHg, and herewith a lower T-cad level, had higher circulating IL-6 and TNF-α concentrations (4.83 vs. 2.00 pg/mL and 9.21 vs. 6.72 pg/mL, respectively) compared to patients with mean a PCWP ≤ 19 mmHg, although the differences did not reach statistical significance.

The power analysis was performed for the finding that T-cad is downregulated in patients with a mean PCWP > 19 mmHg. It reveals that 29 patients in each group are required for the power of analysis to be 80% and the alpha level to be 0.05.

### 3.4. Cardiac T-Cad Levels and Clinical Outcomes

Patients were followed-up for at least 5 years; however, because of early deaths, the median was as follows: 59 months (min. 14 days, max. 94 months). During the follow-up period, 11 (39.2%) patients reached the composite endpoint of the study: 7 (25%) succumbed to cardiovascular causes, 1 (3.6%) received LVAD, and 5 (17.9%) underwent heart transplantation.

**Figure 2:**
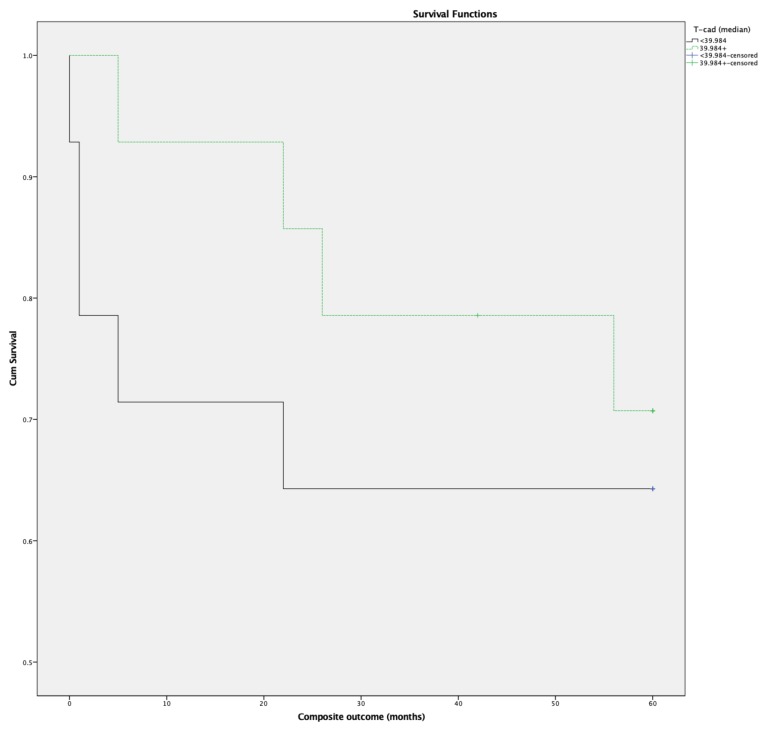
Kaplan–Meier curves categorized by the T-cad median. Five-year follow-up.

The association between the levels of cardiac T-cad and the study outcome was assessed by univariate Cox-regression analysis, revealing no significant association between the two (HR 1.00, *p* = 0.349 CI 95% (0.973–1.026)). The impact of binary T-cad (stratified by mean T-cad 39.984 ng/mg) on patient outcome was also tested. Differences in baseline characteristics of patient groups based on the median T-cad value are depicted in Table S1. Patients with lower cardiac T-cad levels experienced worse outcomes during 5 years of follow-up in the studied cohort (Figure 2).

As the difference between the curves did not reach statistical significance (*p* = 0.528), the data cannot be extrapolated to all patients with NI-DCM and advanced HF. Irrespective of this, the tendency seen would be worthwhile testing in further studies.

## 4. Discussion

To our knowledge, this is the first clinical study evaluating the expression of cardiac T-cad in human myocardium, particularly in patients with NI-DCM and advanced HF, as well as presenting the results of 5 years of follow-up. Recently, downregulation of cardiac APN receptors, including T-cad, was demonstrated in a murine model of progressing HF [47]. As the downregulation of APN receptors was most evident 4 weeks post myocardial infarction, it was assumed that changes in the receptor level might result in cardiac APN resistance at a late stage (such as in chronic HF).

A negative correlation between the levels of cardiac T-cad and the severity of HF, as defined by mean PCWP, was observed in the present study. In addition, a tendency towards lower cardiac T-cad concentration in patients with more pronounced HF (PCWP > 19 mmHg) was also observed. Provided APN association with T-cad is a prerequisite for the physiological activity of APN in the heart [32], diminished T-cad levels in human myocardium might lead to reduced APN signaling as well as a reduced cardioprotective effect. The decreased tissue response to APN was demonstrated in several animal models, despite the elevation of circulating APN levels. Downstream molecules to APN were not activated in a porcine model of DCM [48], and the cardiovascular protective response of APN was abolished in T-cad KO mice [31,32]. Functional APN resistance was also demonstrated in the skeletal muscles of patients with chronic HF [49,50] as well as in the myocardium of patients with advanced HF, which regressed after LVAD implantation [41]. Considering the mutual relationship between circulating APN and tissue T-cad, it is plausible that T-cad negatively regulates the circulating levels of APN, inducing APN clearance from blood and sequestering it to the cardiovascular tissues [51]. The diminished expression of T-cad in patients with HF would lead to higher serum levels of APN and be a supplementary mechanism explaining the “adiponectin paradox” [23].

APN supplementation upregulated T-cad expression in cardiovascular tissues [32,34,52], suggesting that APN signals through T-cad and concomitantly supports the expression of T-cad via a positive feedback loop mechanism [31]. However, no correlation between circulating APN and cardiac T-cad expression was detected in the current study. We suggest that it might be related to the dysfunction of the feed-forward regulation mechanism in advanced HF as it was assumed by Sternberg et al. [53]. On the other hand, the positive relationship between circulating APN and the T-cad documented in the experimental modeling studies of T-cad knockout mice, as well as murine endothelial cell tissue cultures [32,34], might not apply to humans.

A negative correlation between cardiac T-cad levels and the infiltration of myocardial T lymphocytes (CD3+) was detected in the present study. As cardiac inflammation in DCM may lead to LV dysfunction [46] and a worse outcome [54], the higher T-cad levels in the human heart might be associated with the better local control of inflammation, subsequently preserving cardiac function. Having in mind that the binding of T-cad and APN is necessary within myocardium [32], the reduced cardiac T-cad levels might weaken the anti-inflammatory activity of the adipokine.

In the present study, the level of cardiac T-cad, as well as its role in APN-mediated cardioprotection, NI-DCM progression, and the estimation of HF severity, have been investigated. Obtained data show that even if T-cad is not a significant prognostic marker in patients with NI-DCM and advanced HF, it can be an additional biomarker estimating the severity of HF.

The major limitations of this study are a relatively small amount of suitable myocardial biopsy material and the lack of a control group. However, the provided observations might be useful for future hypothesis in larger clinical studies. Further investigations of the molecular mechanisms participating in the development of chronic NI-DCM would help to develop effective therapeutic means preventing further myocardium destruction. It is feasible that T-cad can become a new target for cardioprotective treatment.

## 5. Conclusions

Our findings suggest that reduced cardiac T-cad levels might be an additional indicator of HF severity and lead to the diminished anti-inflammatory role of APN in the myocardium of patients with chronic NI-DCM. T-cad did not prove to be a chronic NI-DCM outcome predictor. Furthermore, further studies are needed in order to extend the role of T-cad in human heart diseases.

## Figures and Tables

**Table 1:** Baseline characteristics of patients.

Variable	Value	No. of Patients
Age (years)	53 ± 8	29
Male/gender, *n* (%)	22 (75.9)	29
BMI (kg/m^2^)	28.8 ± 5.6	29
GFR (ml/min)	104.2 ± 32	25
Duration of symptoms before enrollment (months)	12 (6–60) *	29
NYHA class, *n* (%)		29
II	4 (13.79)	
III	19 (65.52)	
IV	6 (20.69)	
iDCM, *n* (%)	13 (46.4)	28
**Cardiac parameters**		
Permanent atrial fibrillation, *n* (%)	8 (27.59)	29
LVEF (%)	28.28 ± 11.44	29
LVEDD (cm)	6.5 (6.2–7.3) *	29
Average global strain	−9.97 ± 3.67	17
Mean AoP (mmHg)	100 ± 13	27
Mean RAP (mmHg)	11 (7–14) *	29
Mean PCWP (mmHg)	19 (15–30) *	29
Mean PAP (mmHg)	28 (21–38) *	28
**Serum biomarkers**		
BNP (pg/mL)	305 (56.8–1496.2) *	28
Adiponectin (µg/mL)	10.6 (5.30–27.54) *	28
**Markers of inflammation in serum**		
CRP (µg/mL)	2.4 (1.3–11.5) *	27
TNF-α (pg/mL)	8.73 (6.62–9.82) *	28
Il-6 (pg/mL)	2.38 (2–5.14)*	28
**Markers of myocardial immune infiltration (cells/mm^2^)**		
CD3+	9 (7–15) *	28
CD4+	4 (4–6) *	28
CD45ro	6 (5–8) *	28
CD68+	4 (3–5) *	28
**Myocardial adiponectin receptors (ng/mg)**		
T-cadherin	41.160 (22.747–54.338) *	29
Medications used		
Beta blockers, *n* (%)	28 (96.5%)	29
ACE inhibitors, ARB blockers *n* (%)	24 (82.8%)	29
Diuretics and mineralocorticoids receptor blockers, *n* (%)	27 (93.1%)	29
Anticoagulation (atrial fibrillation, EF < 40%), *n* (%)	16 (55.2%)	29
Antiarrhythmic (class III: amiodarone), *n* (%)	4 (13.8%)	29
**Cardiac resynchronization therapy**		
CRT	5 (17.2%)	29
CRTd	1 (3.4%)	29

Data presented as a mean ± SD, a median (interquartile range) *, or *n* (%). Abbreviations: ACE, angiotensin converting enzyme; ARB, angiotensin receptor blockers; AoP, aortic pressure; BMI, Body mass index; BNP, B-type natriuretic protein; CD3+, T cell receptor; CD4+, T helper cell receptor; CD45ro+, memory T cell receptor; CD68+, monocyte/macrophage receptor; CRP, C-reactive protein; CRT, cardiac resynchronization therapy; CRTd, cardiac resynchronization therapy with a defibrillator; GFR, glomerular filtration rate; iDCM, inflammatory dilated cardiomyopathy; IL-6, interleukin-6; LVEDD, left ventricular end-diastolic diameter; LVEF, left ventricular ejection fraction; NYHA, New York Heart Association functional class; PAP, pulmonary artery pressure; PCWP, pulmonary capillary wedge pressure; RAP, right atrial pressure; TNF-α, tumor necrosis factor α.

**Table 2:** Spearman correlations between cardiac T-cad levels and biomarkers of chronic heart failure (HF).

Variable	Rho	*p* Value	No. of Patients
**Serum biomarkers**			
BNP (pg/mL)	−0.013	0.947	27
CRP (μg/mL)	0.354	0.076	26
Adiponectin (μg/mL)	−0.027	0.892	27
IL-6 (pg/mL)	0.185	0.356	27
TNF α (pg/mL)	0.124	0.537	27
**Echocardiographic and hemodynamic parameters**			
LVEF (%)	−0.098	0.621	28
LVEDD (cm)	−0.079	0.689	28
Average global strain	−0.297	0.247	17
Mean AoP (mmHg)	0.015	0.943	26
Mean PCWP (mmHg)	−0.397	0.037	28
Mean PAP (mmHg)	−0.221	0.257	28
Mean RAP (mmHg)	−0.047	0.813	28
**Markers of immune infiltration (cells/mm^2^)**			
CD3+	−0.423	0.028	27
CD4+	0.032	0.874	27
CD45ro	−0.144	0.474	27
CD68+	0.189	0.344	27

One patient with T-cad level as an absolute outlier was omitted from the analysis. Abbreviations: AoP, aortic pressure; BNP, B-type natriuretic protein; CD3+, T cell receptor; CD4+, T helper cell receptor; CD45ro+, memory T cell receptor; CD68+, monocyte/macrophage receptor; CRP, C-reactive protein; IL-6, interleukin-6; TNF-α, tumor necrosis factor α; LVEDD, left ventricular end-diastolic diameter; LVEF, left ventricular ejection fraction; PAP, pulmonary artery pressure; PCWP, pulmonary capillary wedge pressure; RAP, right atrial pressure.

**Table 3:** Differences in patient groups based on the mean PCWP.

Differences in Patient Groups Based on the Mean PCWP					
	Mean PCWP ≤ 19 mmHg		Mean PCWP > 19 mmHg		
Variable	Median (IQR)	No. of Patients	Median (IQR)	No. of Patients	*p* Value
Age (years)	52 (48–54)	15	53 (48–58)	13	0.474
Male gender, *n* (%)	11 (73.3)	15	10 (76.9)	13	1
BMI (kg/m^2^)	28 (25.3–34.9)	15	28 (25.2–31.8)	13	0.872
GFR (ml/min)	103.7 (91.5–119.7)	14	104 (74.8–132.9)	10	0.931
NYHA class, *n* (%)		15		13	0.731
II	3 (20.0)		1 (7.7)		
III	10 (66.7)		9 (69.2)		
IV	2 (13.3)		3 (23.1)		
iDCM, *n* (%)	6 (46.2)	14	6 (42.9)	13	1
**Echocardiographic parameters**					
LVEF (%)	35 (30–42)	15	20 (17-30	13	0.003
Average global strain	−12.46 (−13.377–9.583)	10	−8.54 (−9.45–5.15)	7	0.007
**Serum biomarkers**					
BNP (pg/mL)	75 (30.75–304.7)	15	1134 (335.8–2653.4)	12	0.005
CRB (µg/mL)	3.0 (1.2–13.3)	14	3.3 (1.4–9.45)	12	0.959
Adiponectin (μg/mL)	6.6 (5.1–16.3)	15	14.9 (7.73–27.57)	12	0.217
IL-6 (pg/mL)	2.00 (2–3.3)	15	4.83 (2–14.44)	12	0.056
TNF-α (pg/mL)	6.72 (6.01–9.6)	15	9.21 (8.65–10.61)	12	0.075
**Cardiac inflammatory infiltration markers (cells/mm^2^)**					
CD3+	9 (7–11)	14	8 (7–17)	13	0.450
CD4+	5 (4–6)	14	4 (3–6)	13	0.472
CD45ro	6 (4–8)	14	6 (5–7)	13	0.961
CD68+	5 (2–5)	14	3 (3–5)	13	0.920
**Myocardial adiponectin receptors (ng/mg)**					
T-cadherin	45.654 (32.184–65.583)	15	26.805 (15.926–50.362)	13	*0.058*
**Medications used**					
Beta blockers, *n* (%)	15 (100)	15	13 (100)	13	
ACE inhibitors, ARB blockers *n* (%)	13 (86.7)	15	10 (76.9)	13	0.639
Diuretics and mineralocorticoids receptor blockers, *n* (%)	14 (93.3)	15	12 (92.3)	13	1
Anticoagulation (atrial fibrillation, EF < 40%), *n* (%)	6 (40)	15	10 (76.9)	13	0.049
Antiarrhythmic (class III: amiodarone), *n* (%)	1 (6.7)	15	3 (23.1)	13	0.311

Data presented as a median and an IQR (interquartile range). Significant differences are bolded (Wilcoxon rank sum test or χ^2^ test for equality of proportions). Significant at the *p* level 0.05 (2-tailed). One patient with T-cad level as an absolute outlier was omitted from the analysis. Abbreviations: ACE, angiotensin converting enzyme; ARB, angiotensin receptor blocker; BNP, B-type natriuretic protein; CD3+, T cell receptor; CD4+, T helper cell receptor; CD45ro+, memory T cell receptor; CD68+, monocyte/macrophage receptor; CRP, C-reactive protein; IL-6, interleukin-6; LVEF, left ventricular ejection fraction; TNF-α, tumor necrosis factor α.

## Supplementary Materials

The following are available online at https://www.mdpi.com/1010-660X/56/1/27/s1, Table S1: Differences in patient groups based on T-cad median.
