# Supplementary material for: The Role of Cardiac T-Cadherin in the Indicating Heart Failure Severity of Patients with Non-Ischemic Dilated Cardiomyopathy
Source: Medicina (Kaunas). 2020 Jan 9;56(1):27. doi: 10.3390/medicina56010027 (PMC7023024; doi:10.3390/medicina56010027)
Supplement: Supplementary file 1 [file medicina-56-00027-s001.zip › Supplement_table_1.docx]

**Table S1. Differences in patient groups based on T-cad median**

|  | **T-cad =<39.984 ng/mg** | | **T-cad >39.984 ng/mg** | | **p value** |
| --- | --- | --- | --- | --- | --- |
| **Variable** | **Median (interquartile range)** | **No. of pts.** | **Median (interquartile range)** | **No. of pts.** |  |
| Age (years) | 53 (50-58) | 14 | 50.5 (46-55) | 14 | 0.322 |
| **Serum biomarkers** | | | | | |
| BNP (pg/mL) | 381 (74.8-1793.6) | 13 | 160 (38.2-764.1) | 14 | 0.43 |
| CRB (µg/mL) | 1.8 (1.1-4.2) | 13 | 6.7 (1.5-20.6) | 13 | 0.106 |
| Adiponectin (μg/ml) | 10.8 (6.5-15.3) | 13 | 7.3 (4.3-30.71) | 14 | 0.867 |
| IL-6 (pg/mL) | 2.00 (2-4.62) | 13 | 2.38 (2-4.86) | 14 | 0.8 |
| TNF α (pg/mL) | 8.47 (6.72-9.80) | 13 | 8.94 (6.62-9.74) | 14 | 0.981 |
| **Echocardiographic and hemodynamic parameters** | | | | | |
| LVEF (%) | 30 (21-34) | 14 | 30 (20-39) | 14 | 0.889 |
| LV average global strain | -8.97 (-10.41  -6.14) | 8 | -12.25 (-13.59  -9.01) | 9 | 0.093 |
| Mean AoP (mmHg) | 97 (90-107) | 12 | 101 (100-104) | 14 | 0.587 |
| Mean PCWP (mmHg) | 23 (18-31) | 14 | 16 (13-21) | 14 | 0.062 |
| Mean PAP (mmHg) | 34 (28-38) | 14 | 24 (21-34) | 14 | 0.112 |
| Mean RAP (mmHg) | 10 (7-16) | 14 | 10 (6-13) | 14 | 0.612 |
| **Markers of immune infiltration (cells/mm2)** | | | | | |
| CD3+ | 13 (8-17) | 14 | 7 (5-8) | 13 | **0.005** |
| CD4+ | 5 (4-6) | 14 | 4 (3-6) | 13 | 0.503 |
| CD45ro+ | 7 (5-8) | 14 | 5 (3-8) | 13 | 0.13 |
| CD68+ | 4 (2-5) | 14 | 4 (3-5) | 13 | 0.67 |
| **Myocardial adiponectin receptors** | | | | | |
| Adipo R1 (ng/mg) | 23.220 (17.346-30.641) | 12 | 30.830 (28.808-51.708) | 8 | 0.098 |

Data are presented as median (interquartile range). Significant differences are bolded (Wilcoxon rank sum test). Significant at the p level 0.05 (2-tailed).

Abbreviations: Adipo R1, adiponectin receptor 1; AoP, aortic pressure; BNP, B-type natriuretic protein; CD3+, T cell receptor; CD4+: T helper cell receptor; CD45ro+, memory T cell receptor; CD68+, monocyte/macrophage receptor; CRP, C-reactive protein; IL-6, interleukin-6; LVEF, left ventricular ejection fraction; PAP, pulmonary artery pressure ; PCWP, pulmonary capillary wedge pressure; RAP, right atrial pressure, TNF-α, tumor necrosis factor α.
